# Salmagundi

**Published:** 1884-03

**Authors:** 


### SALMAGUNDI.

EDITED BY E. G. BETTY, D.D.S.

THE following is said to be a legitimate word, though we can not find it in any of the dictionaries:

Infantrieleibregimentskasernenarrestantenaufsichtsunteroffizierszimmernummer

GALVANIZED iron pails for drinking water should not be used. The zinc coating is readily acted upon by water, forming a poisonous oxide of zinc.—*Scientific American.*

OPERATING BY THE ELECTRIC LIGHT.—Iridectomy and other delicate operations about the eye were recently performed in London under the electric light. It proved very satisfactory.

TAR may be readily removed from the hands by rubbing with the outside of fresh orange or lemon peel, and wiping dry immediately. The volatile oils in the skins dissolve the tar, so that it can be wiped off.—*Scientific American.*

---

IMITATION AMBER.—Roessler's recipe is to melt one part of rosin (colophonium), then add two parts, by weight, of shellac. When the mixture becomes sufficiently fluid one part of white rosin, that should be clear as water, is then added.—*Exchange.*

---

FROM a large list of administrations of anæsthetics, reaching over many thousands of cases, both in America and Great Britain, the average risk to life has been pretty accurately determined. It amounts to about one death in 2,800 administrations.

---

" A London authority on the effects of tea and coffee calls tea the "tobacco of women," but it requires less digestive power than coffee, which, however, is a better stimulant, and one that is a good substitute for spirits."

---

A number of toothless individuals in Iowa are out gunning for a traveling dentist who pulled out their teeth free, and collected half the price of new sets which he forgot to bring around —*Daily Paper.*

---

THE tendons of the tail of the kangaroo can be easily split into threads two feet in length, rivaling silk in strength, softness, fineness, beauty of color, and finish. Such tendons for ligatures and sutures, promise to supplant silk almost entirely in surgery. —*Ibid.*

---

THE finding of sets of false teeth in ancient graves in the Santa Barbara Islands, on the Pacific Coast, should stimulate endeavor in the line of further investigation. There may be turned up a bang, a palpitator, or a bustle or two, to show the drift of fashion in those days.—*Daily Paper.*

The Boston *Medical and Surgical Journal* raises its voice against any attempt on the part of the adulterators of drugs, to obtain a repeal of the present law, which is so essential "for the protection of the public at large and of the medical profession in the discharge of its duties to the public."

A consignment of very lively leeches was among the first day's receipts at the General Postoffice in London on the inauguration of the new parcels post. The box containing them was a very slight one, and becoming fractured in transit, the contents escaped, and traversed the establishment in search of a promising "subject."

It is curious that the best dental appliances are made in America, or at any rate have originated there. In fact, dental surgery is even a more extensive profession in America than it is anywhere else; and this is said to be, we know not with what truth, because no other people have such bad teeth as the Americans.—*Glasgow Herald.*

Decoloration.—"It is very desirable, for some purposes, to have tincture of iodine decolorized. Various agents have been used for this purpose. In addition to those we have used Listerine may be employed. About an equal quantity of Listerine to the officinal tincture of iodine will, within a day or two, render it entirely colorless, and, apparently without impairing its action. Try it and report."

The hyposulphite of soda will perfectly decolorize tinct. iodine, and without impairing its qualities.

"We shall, perhaps, hear of rioting among the old fashioned tooth carpenters, and of efforts to break down new systems designed to reform the jaw-breaking methods. For example, here comes a Swiss dentist with a new process of tooth-drawing. A small square of india-rubber, pierced with a central hole, is pushed over the tooth till the upper part of the root is reached. The india-rubber gradually contracts, pulls on the root, and the offending tooth is finally enucleated, without causing the patient any pain whatever. Four or five days are generally required to complete the operation. Very slight bleeding and a slight swelling of the gum are the only inconveniences experienced."

### DARWINISM.

The monkey climbed toward the raging sky
  And twisted his tail 'round a lofty limb,
While the flood beneath went thundering by,
  For he was a monkey that couldn't swim.
But the man was caught in the torrents mad,
  And his dying speech in these words ran:
"If I had a tail as my forefathers had,
  I'd be a live monkey and not a drowned man."

—*Anonymous.*

This is *apropos* during the flood at present throughout the Ohio Valley.

### A "FAST" YOUNG LADY.

ASHLAND, KY., January 15, 1884.

*Editor Salmagundi:*

I met with a patient a day or two since—*ten years and one month old*—who had shed all her temporary teeth; the permanent ones being fully in place and two of the second molars completely erupted. Some of the teeth also were badly decayed, the pulp of one of the centrals being dead. Have you often met cases of greater precocity? Yours inquiringly,

D. S. DIBBLE, D.D.S.

We have not. If any of our readers have we shall be glad to hear from them.

### SUCCESSFUL DENTAL OPERATION ON FISH.

Mr. Hugo Mulertt, the pisiculturist of this city, sends us the following extract from an article in the *Aquarius:*

"Seth Green, the well-known fish culturist, kept several California mountain trout in an aquarium. These otherwise tame fish would frequently fight with one another, often inflicting ghastly wounds upon each other that might sooner or later result in death. To prevent this, it was determined to extract their teeth, as these organs are mostly used as weapons of offense and defense. The operation was successfully performed, and the fish suffered no serious consequences, as the loss of the teeth as prehensile organs was more than supplied by the facility with which fish confined in an aquarium can be fed."

---

### SUPERSTITIOUS LEGEND.

We are told that when St. Helena, of pious memory, had discovered the true cross of Christ she permitted various fragments to be taken from it, which were incased, some in gold, and some in gems, and conveyed to Europe, leaving the principal or main part of the wood in the charge of the Bishop of Jerusalem, who exhibited it annually at Easter, until Chosroes, King of Persia, plundered Jerusalem in the reign of the Emperor Phocas, and took away this holy relic.

Before this fatal event we are taught to believe by Rigordus, an historian of the thirteenth century, that the mouths of Christians used to be supplied with thirty; or, in some instances, no doubt according to their faith, with thirty-two teeth; but that, *after* the cross was stolen by the infidels, no mortal has ever been allowed more than twenty-three.—*Ten Thousand Wonderful Things.*

---

### A DANGEROUS CHEMICAL EXPERIMENT.

An event of considerable interest occurred in the chemical department of Amherst College, Saturday. Once in three years the experiment is made of condensing carbonic dioxide. So difficult and dangerous is the undertaking by this process that it is forbidden by law in all countries except in the United States, and probably Amherst is the only college where it is undertaken. Two iron cylinders are used, one the generator, the other the receiver.

They resemble howitzers fitted with strong iron bands and peculiar valves. Bicarbonate of soda and sulphuric acid are placed in the generator in such a way as not to mingle until the cylinder is securely closed. The union of the substances generates carbonic acid gas with terrific pressure (being about a ton to every four square inches), and this passes into the receiver, which is packed in ice and salt. The process is repeated twelve times, until the gas in the receiver is forced by pressure and cold into liquid form. When this is allowed to flow out it evaporates so rapidly that it forms a solid, snow-like mass, having the surprising temperature of 140° below zero.

Mercury poured upon it freezes instantly, and the effect of touching it is about the same as handling a red-hot coal. The great danger in the experiment arises from the tremendous pressure—and thus the liability of a bursting cylinder. The experiment Saturday, which was in charge of Instructor Pond and the senior chemistry division, was of great interest to the entire college.—*Springfield Republican.*

---

REPAIRING PLATES OF WATT'S METAL.

Not long ago in reading the printed directions for using the above metal we noticed that instructions were given for repairing plates by *pouring.* Now, we respect Dr. Watt very much, and like his new metal nearly as well, but would like to whisper in his ear that pouring is not *always* the best thing to do. We greatly prefer a tinner's soldering iron, for by its use we need no investment, and save that much time. In one case, the flask had been poured just as a meddlesome matty came into the laboratory; curiosity prompted him to look at the flask and then pick it up, the result being that a quantity of the metal escaped from a point corresponding with the joint between the centrals. After cooling and separation, a portion of each heel was gone and part way upon the sides of the molar blocks. The piece was banked with plaster, the surfaces of the metal freed from oxide and moistened with chloride of zinc, heated up to a point just this side of melting, the surfaces again moistened with the flux and the repair metal poured in. No attachment took place even after repeated trials. Muriatic acid c. p. was then used as a flux, and the soldering iron applied as a vehicle to add the melted metal bit by bit until the lost portion was restored. Upon filing and polishing no trace of union could be found. But be *very* careful with the iron. For attaching a new block, bank it up with plaster from the front and flow in the metal from the iron until the pins are tinned and the space filled; the *best* flux being the strong acid. Of course it helps very materially to heat up the piece. If Dr. Watt has any objection to this method of repair we are open to conviction. Very likely the method is not new, as we have an indistinct recollection of having heard of the soldering iron before, but merely give it as a preference, the only objection urged against it being the great liability to melt down the plate. This need not happen with any one who knows how to mend a hole in a tin can.

